# Role of thrombin receptor in breast cancer invasiveness

**DOI:** 10.1038/sj.bjc.6690063

**Published:** 1999-02

**Authors:** K P Henrikson, S L Salazar, J W Fenton II, B T Pentecost

**Affiliations:** Wadsworth Center, New York State Health Department, Albany, NY, 12201, USA; Department of Biomedical Sciences, State University of New York at Albany, Albany, NY, 12201, USA

**Keywords:** thrombin, breast cancer, thrombin receptor, invasive cancer, cell culture

## Abstract

Invasion, the ability of an epithelial cancer cell to detach from and move through a basement membrane, is a central process in tumour metastasis. Two components of invasion are proteolysis of extracellular matrix and cellular movement through it. A potential promoter of these two processes is thrombin, the serine proteinase derived from the ubiquitous plasma protein prothrombin. Thrombin promotes the invasion of MDA-MB231 breast tumour cells (a highly aggressive cell line) in an in vitro assay. Invasion by MDA-MB436 and MCF-7 cells, less aggressive cell lines, is not promoted by thrombin. Thrombin, added to the cells, is a stimulator of cellular movement; fibroblast-conditioned medium is the chemotaxin. Thrombin-promoted invasion is inhibited by hirudin. Stimulation of invasion is a receptor-mediated process that is mimicked by a thrombin receptor-activating peptide. Thrombin has no effect on chemotaxis in vitro. Thrombin receptor is detectable on the surface of MDA-MB231 cells, but not on the other two cell lines. Introduction of oestrogen receptors into MDA-MB231 cells by transfection with pHEO had no effect on thrombin receptor expression, in the presence or absence of oestradiol. This paper demonstrates that thrombin increases invasion by the aggressive breast cancer cell line MDA-MB231 by a thrombin receptor-dependent mechanism. © 1999 Cancer Research Campaign


*British Journal of Cancer* (1999) **79**(3/4), 401–406
© 1999 Cancer Research Campaign
Article no. bjoc.1998.0063

### Role of thrombin receptor in breast cancer invasiveness

**KP Henrikson[1,2], SL Salazar[1,2], JW Fenton II[1] and BT Pentecost[1,2]**

[1]Wadsworth Center, New York State Health Department, Albany, NY 12201, USA; [2]Department of Biomedical Sciences, State University of New York at Albany, NY 12201, USA

**Summary** Invasion, the ability of an epithelial cancer cell to detach from and move through a basement membrane, is a central process in tumour metastasis. Two components of invasion are proteolysis of extracellular matrix and cellular movement through it. A potential promoter of these two processes is thrombin, the serine proteinase derived from the ubiquitous plasma protein prothrombin. Thrombin promotes the invasion of MDA-MB231 breast tumour cells (a highly aggressive cell line) in an in vitro assay. Invasion by MDA-MB436 and MCF-7 cells, less aggressive cell lines, is not promoted by thrombin. Thrombin, added to the cells, is a stimulator of cellular movement; fibroblast-conditioned medium is the chemotaxin. Thrombin-promoted invasion is inhibited by hirudin. Stimulation of invasion is a receptor-mediated process that is mimicked by a thrombin receptor-activating peptide. Thrombin has no effect on chemotaxis in vitro. Thrombin receptor is detectable on the surface of MDA-MB231 cells, but not on the other two cell lines. Introduction of oestrogen receptors into MDA-MB231 cells by transfection with pHEO had no effect on thrombin receptor expression, in the presence or absence of oestradiol. This paper demonstrates that thrombin increases invasion by the aggressive breast cancer cell line MDA-MB231 by a thrombin receptor-dependent mechanism.

**Keywords:** thrombin; breast cancer; thrombin receptor; invasive cancer; cell culture

The major cause of mortality in breast cancer is the progression of metastatic disease. Although loss of oestrogen receptors (ER) and progesterone receptors is associated with more aggressive disease, 30% of patients with receptor-positive, node-negative tumours develop metastases (Price et al, 1997). A component of the metastatic process is invasion, which is accomplished when the cells can transit the extracellular matrix (ECM) layer. More accurate prognoses might be possible if factors could be identified that either promote invasion or are markers for it, independent of steroid receptor status. Specific treatments to prevent invasion might also be developed.

Thrombin is a growth-regulatory protein at nanomolar concentrations. Thrombin is also a serine proteinase that is generally associated with coagulation. Its proenzyme, prothrombin, occurs in the plasma at concentrations up to 3 μM (Shapiro and McCord, 1978). Thrombin and its associated receptor, TR-1, are involved in the activation of platelets (McNicol and Gerrard, 1993; Vittet and Chevillard, 1993), growth stimulation of immature rat uterine stromal cells (Arena et al, 1996), alteration of permeability in vascular endothelial cells (DeMichele and Minnear, 1992; Garcia et al, 1995), DNA synthesis in astrocytoma cells (Aragay et al, 1995) and endothelial cell growth in angiogenesis (Fidler and Ellis, 1994). Thrombin also has been implicated in tumour progression (Wojtukiewicz et al, 1995).

The initial step in characterization of thrombin's role in breast tumour cell invasion is a description of the presence of TR-1 on invasive breast tumour cell lines and of thrombin's influence on in vitro invasion by those cell lines. We report the presence of TR-1 and activity of thrombin in stimulating invasion by the MDA-MB231 breast cancer cell line and its absence in other, less aggressive, breast tumour cell lines.

*Received 18 March 1998*
*Revised 7 July 1998*
*Accepted 13 July 1998*

*Correspondence to:* KP Henrikson, Wadsworth Center, Empire State Plaza, PO Box 509, Albany, NY 12201, USA

### MATERIALS AND METHODS

### Cell culture

Cells were cultured in Dulbecco's modified Eagle medium (DMEM) without phenol red, with streptomycin, penicillin, non-essential amino acids, glutamine and 5% serum (Hyclonedefined calf serum for MDA-MB231 and MDA-MB436; Gibco fetal calf serum for MCF-7 cells).

Conditioned medium was prepared by incubating confluent 3T3 cultures with bovine serum albumin (BSA) medium (DMEM without phenol red, containing 0.1% BSA and antibiotics) for 24 h. Conditioned medium was stored frozen and was centrifuged immediately before use to remove cells and debris.

### Invasion assays

Invasion chambers with 8-μm pores were purchased from Becton–Dickinson. Chambers were used without modification for chemotaxis assays. Matrigel-coated chambers were used for invasion assays. Matrigel, purchased from Becton–Dickinson, was diluted in ice-cold DMEM without additions. One hundred microlitres (38 μg) was pipetted into each chamber in a 24-well plate. The plate was incubated at 37°C for 1 h, then medium was removed by aspiration and replaced with 400 μl of DMEM. Chambers were either used immediately or after overnight storage at 4°C.

Assays were performed in 24-well plates. The wells contained 0.75 ml of either BSA medium (for blanks) or conditioned medium. Inserts contained cells in BSA medium. Ten thousand MDA-MB231 or MDA-MB436 cells, or 50 000 MCF-7 cells in BSA medium with appropriate additions were placed in the inserts. Media and cells were removed from the wells after 24 h at 37°C for studies of invasion, or 6 h at 37°C for studies of chemotaxis,

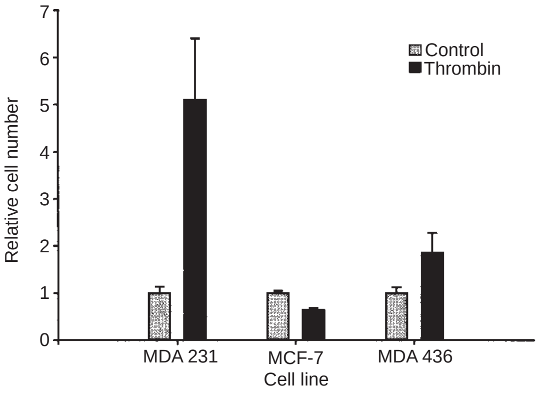

**Figure 1** Modulation of invasion by cell lines upon treatment with 100 nM thrombin. Six to nine independent experiments were performed with each cell line; four replicates of each treatment were carried out. In the controls, the actual mean number of invading cells (± s.e.m.) was 317 ± 136 for MDA-MB231 cells, 51 ± 9 for MCF-7 cells, and 217 ± 119 for MDA-MB436 cells. For comparative purposes, the number of invading cells in the controls was restated as 1.0 (± relative s.e.m.). Invasion of thrombin-treated cells is given relative to controls. By Student's *t* test, $P = 0.004$ for stimulation of invasion of MDA-MB231 cells by thrombin; thrombin does not significantly stimulate the other cell lines

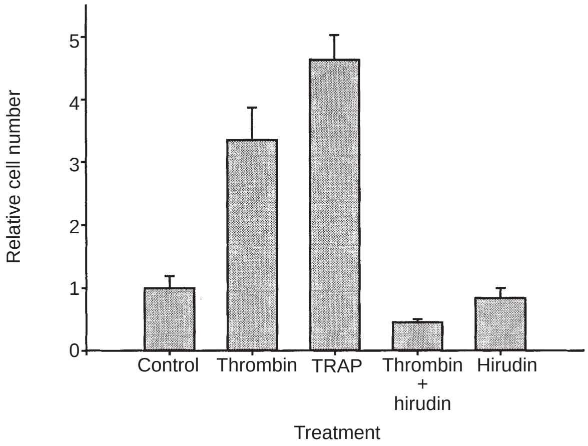

**Figure 3** Effects of hirudin and TRAP on invasion by MDA-MB231 cells. Thrombin (100 nM), 50 μM TRAP, and 140 nM hirudin were used. Each bar represents the sum of at least two independent experiments with three or four replicates of each point. The actual number of invading cells in the control was 210 ± 114. Thrombin and TRAP both significantly increased invasion, $P < 0.001$ compared with control for each

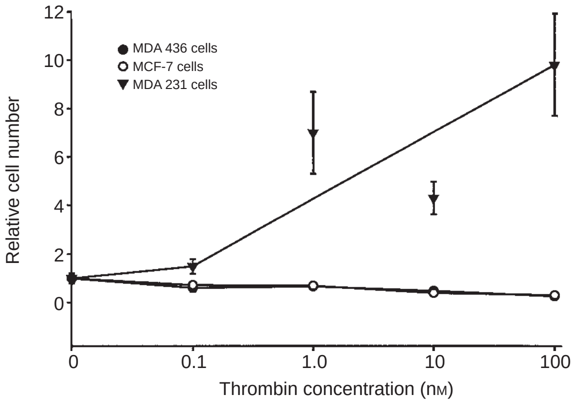

**Figure 2** Concentration dependence of stimulation of invasion by thrombin. Each line represents the sum of two experiments with four replicates of each point. The mean number of cells invading in the control samples was: MDA-MB231 cells, 501 ± 217; MDA-MB436 cells, 750 ± 160; MCF-7 cells, 79 ± 26

leaving cells that had migrated on the underside of the membrane. Membranes were stained with Diff Quik, dried and analysed with the NIH Image program. About 20% of the total membrane area was counted. Each experimental point had four replicates.

### Flow cytometry

Cells were harvested for flow cytometry by scraping into BSA medium plus protease inhibitors (aprotinin 1.6 U $ml^{-1}$, soybean trypsin inhibitor 2 μg $ml^{-1}$, leupeptin 2 μg $ml^{-1}$, pepstatin 7 μg $ml^{-1}$, antipain 37 μg $ml^{-1}$, bestatin 0.1 μg $ml^{-1}$). Cells were washed once with the same medium, counted, washed with phosphate buffered saline–0.1% azide (PBSA), and diluted for assay in PBSA. Aliquots of $10^6$ cells were incubated with primary antibody, washed twice with PBSA, incubated with FITC-labelled goat anti-mouse (IgG and IgM) (Boehringer–Mannhein), washed twice and analysed in a FACScan instrument (Becton–Dickinson).

The primary antibody, PF-11, is a monoclonal raised against a peptide containing residues 29–69 of the human TR-1 (Kranzhofer et al, 1996), which includes the thrombin binding site and the anion-binding exosite of TR-1. It was generously provided by Dr Shaun Coughlin, UCSF. A mouse IgG, 2b (Sigma), was the isotype control.

### Stable transfection with pHEO

MDA-MB231 cells were stably transfected with pHEO to form the oestrogen receptor-positive cell line MDAL3, as previously described (Tora et al, 1989; Pentecost, 1998).

### Statistics

Results are presented as means ± s.e.m. Data were analysed by Student's two tailed test or by ANOVA with the Student–Newman–Keuls post test for differences among groups.

### RESULTS

### Thrombin-stimulated invasion through Matrigel

The three cell lines have been characterized in the literature as highly invasive (MDA-MB231), moderately invasive (MDA-MB436), and minimally invasive (MCF-7) (Thompson et al, 1992). The relative invasiveness of the cell lines was confirmed as shown in Figure 1. The extent of thrombin-stimulated invasion is given relative to control values. The broad differences in the number of migratory cells in control samples, 51 for MCF-7 cells, 217 for MDA-MB436 cells, and 317 for MDA-MB231 cells, emphasize the very different inherent invasive capacities of these cell lines on a Matrigel matrix. The effect of 100 nM thrombin on invasion is also given. Thrombin stimulated invasion in the MDA-MB231 cell line, but did not stimulate MDA-MB436 or MCF-7 cells.

Thrombin is a stimulator of movement, not a chemoattractant, in invasion assays. The chemoattractant is the 3T3-conditioned medium in the well, whereas the thrombin is placed in the insert with the cells.

*© Cancer Research Campaign 1999*

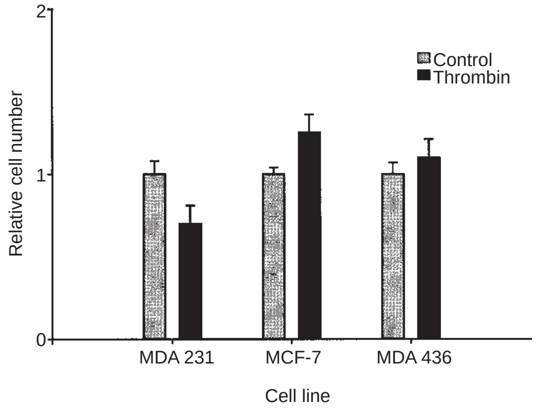

**Figure 4** Chemotaxis by cell lines with 100 nM thrombin. Four or five independent experiments were carried out with each cell line, with four replicates of each treatment. The actual mean number of stained cells in the controls was 752 ± 81 for MDA-MB231, 25 ± 7 for MCF-7, and 165 ± 61 for MDA-MB436 cells. The control and thrombin values are not statistically different except for MCF-7 cells, in which thrombin stimulated chemotaxis from 25 cells to 30, $P = 0.036$

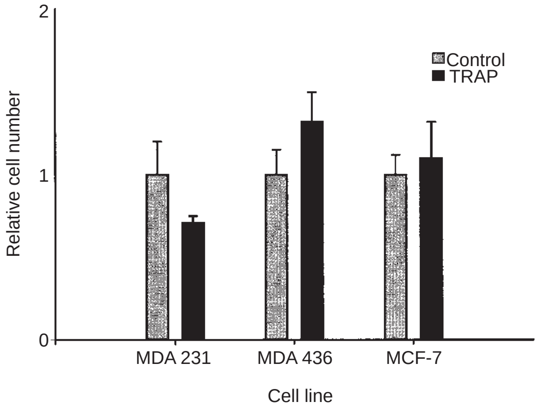

**Figure 5** Chemotaxis and TRAP. Two or three independent experiments were carried out with each cell line, with four replicates of control samples and four to six replicates of TRAP samples. The actual mean number of stained cells in the controls was 1068 ± 160 for MDA-MB231 cells, 138 ± 32 for MDA-MB436 cells, and 18 ± 1.8 for MCF-7 cells. There was no effect of 50 μM TRAP on chemotaxis

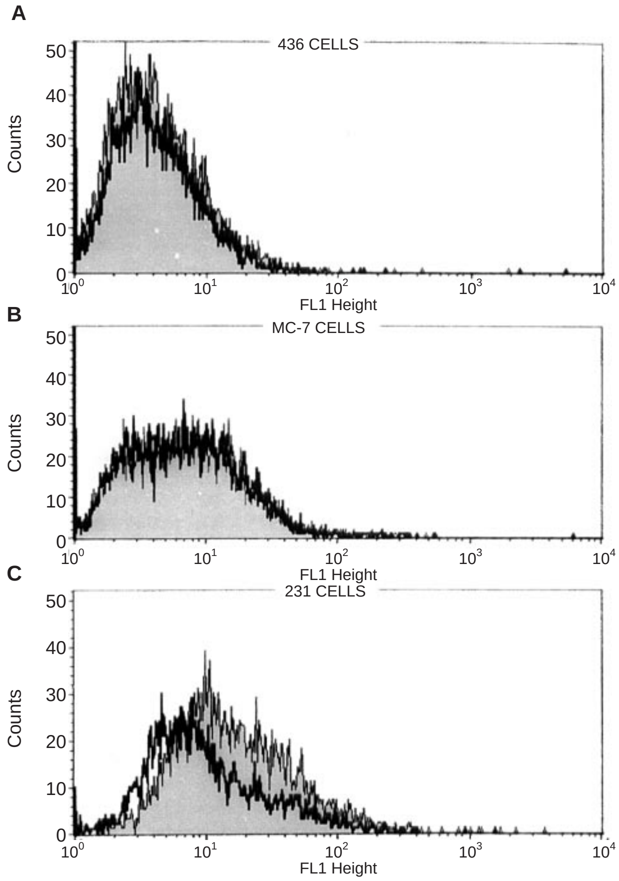

**Figure 6** Detection of TR-1 on cell lines by flow cytometry. Cells were labelled as described in the Materials and methods section and analysed by FACScan. Typical scans are shown for MDA-MB436 cells (**A**), MCF-7 cells (**B**), and MDA-MB231 cells (**C**). The relative fluorescence intensity when cells were labelled with the isotype control is given by the open line. The solid peak gives the relative fluorescence intensity of the cells labelled with antibody to TR-1. Only for MDA-MB231 cells was there a difference between the two peaks

The concentration dependence of thrombin stimulation is shown in Figure 2. Data are reported relative to invasion by the unstimulated cell cultures. MDA-MB231 cells were stimulated by all concentrations above 0.1 nM. MDA-MB436 cells and MCF-7 cells were not stimulated by any concentration of thrombin, up to 100 nM. Thrombin had no effect on the viability of the cell lines (data not shown).

Actions of thrombin can be inhibited by hirudin, a highly specific inhibitor of proteolysis by thrombin. Effects that occur through the thrombin receptor, TR-1, require enzymatically active thrombin and can be elicited by thrombin receptor activating peptide (TRAP). Thrombin-stimulated invasion by MDA-MB231 cells, shown in Figure 3, was completely eliminated by a slight molar excess of hirudin (140 nM hirudin and 100 nM thrombin), whereas hirudin alone had no effect. Similar results (not shown) were obtained with thrombin inactivated with the highly specific thrombin inhibitor, Pro–Phe–Arg chloromethyl ketone (PPACK). Stimulation of invasion by 50 μM TRAP, also shown in Figure 3, demonstrated the role of TR-1 in thrombin action. No stimulation occurred with an unrelated peptide, TCHDVLNETLLHG, not shown.

### Effect of thrombin on chemotaxis

Movement of cells through the uncoated membrane of an invasion chamber (chemotaxis) is a measure of cell motility, independent of attachment to or digestion of ECM. Figure 4 shows that 100 nM thrombin had no effect on chemotaxis except in MCF-7 cells where it increased the number of migrating cells from 25 to 30.

The absence of a TR-1-mediated effect on chemotaxis was confirmed by experiments with TRAP. At 50 μM, TRAP had no effect on chemotaxis for any of the three cell lines, as shown in Figure 5.

A failure of thrombin to stimulate chemotaxis would result if the thrombin diffused through the open pores of the invasion chamber and digested the chemoattractant in the well. To eliminate that possibility, 3T3-conditioned medium was pretreated with thrombin and the excess thrombin was inactivated with PPACK. The treated media were then used as the chemoattractant for invasion assays with MDA-MB231 cells. If thrombin digested the chemotaxin, the treated media would not stimulate invasion.

© Cancer Research Campaign 1999

**Table 1** Flow cytometry for TR-1 on breast tumour cell lines. The indicated number of samples was analysed in five to seven independent experiments. Data are given as means ± s.e.m. Isotype control and TR-1 antibody (PF-11) were used at the same protein concentration

| Cell line | Sample number | Relative fluorescence intensity | | Ratio PF-11/control | *P*-value |
|---|---|---|---|---|---|
| MDA-MB231 | 14 | Control | 3.85 ± 1.08 | 1.45 ± 0.31 | <0.001 |
| | | PF-11 | 5.52 ± 0.66 | | |
| MDA-MB436 | 10 | Control | 6.41 ± 2.10 | 1.05 ± 0.26 | 0.57 |
| | | PF-11 | 7.00 ± 3.69 | | |
| MCF-7 | 13 | Control | 5.77 ± 1.18 | 0.98 ± 0.22 | 0.79 |
| | | PF-11 | 5.52 ± 1.07 | | |

**Table 2** Flow cytometry for TR-1 on MDA-MB231 cells transfected with oestrogen receptor (MDAL3 cells). The indicated number of samples was analysed in two independent experiments. Data are given as means ± s.e.m. Cells were grown to confluence in the presence or absence of 10 nM oestradiol for 5–7 days. Fresh oestradiol was added two or three times, with each change of medium

| Cell treatment | Sample number | Ratio PF-11/control | *P*-value |
|---|---|---|---|
| No oestradiol | 14 | 1.26 ± 0.05 | <0.001 compared with control |
| Oestradiol ($10^{-9}$ M) | 4 | 1.17 ± 0.05 | <0.001 compared with control<br>*P* = 0.08 (not significant) compared with cells grown without oestradiol |

Media treated with thrombin and PPACK or PPACK only stimulated 50% of the invasion stimulated by control media. Addition of thrombin to the cells (50 nM) resulted in a two- to threefold stimulation of invasion. The absence of an effect of thrombin on chemotaxis cannot be attributed to destruction of the chemotaxin.

### TR-1 on cell surfaces

To demonstrate TR-1 on the surface of the three cell lines, cells were harvested by scraping, labelled with monoclonal antibody PF-11 for TR-1, and analysed by flow cytometry. The results of a typical determination are shown in Figure 6. For both MCF-7 and MDA-MB436 cells, the PF-11-labelled peak coincided with the isotype control, indicating the absence of detectable TR-1 on the surface of these cell lines. The MDA-MB231 cells had a slightly shifted peak with PF-11, indicating the presence of TR-1 on these cells. Because the differences between control and PF-11-treated cells were small, these experiments were repeated five to seven times with duplicate samples. Averaged data, shown in Table 1, were subjected to statistical analysis, which gives validity to the detection of TR-1 on MDA-MB231 cells.

### Oestrogen receptor and TR-1 expression

To investigate whether a link exists between oestrogen and TR-1, the MDAL3 cell line, derived from MDA-MB231 cells by stable transfection with the pHEO oestrogen receptor construct (Tora et al, 1989; Pentecost, 1998), was used. Cells were grown with or without 10 nM oestradiol for 5–7 days until confluence was reached. TR-1 was assayed by flow cytometry. As shown in Table 2, TR-1 was present in the MDAL3 cells; there was no difference in the amount of TR-1 detected in the cells whether oestradiol was present or not.

### DISCUSSION

Thrombin has long been thought to play a role in cancer biology. Activation of coagulation in cancer patients was first observed in 1865 (Wojtukiewicz et al, 1993); in the 1930s, it was suggested that coagulation factors might contribute to tumour growth (Zacharski, 1996). Anti-thrombotic therapy with warfarin or with heparin has been beneficial in the treatment of small-cell lung carcinoma (Zacharski et al, 1992; Zacharski and Costantini, 1995).

Understanding of the cellular role of thrombin in tumour biology is incomplete. Zacharski et al (1995) have demonstrated the presence of thrombin by immunohistochemistry on cells of small-cell lung carcinoma, renal carcinoma and melanoma. Nierodzik et al (1996) demonstrated the presence of TR-1 on the surface of six (non-breast) tumour cell lines and showed that receptor activation increased platelet adhesion and tumour tyrosine phosphorylation. Wojtukiewicz et al (1993) showed that thrombin enhanced tumour cell adhesion to endothelial cells and to fibronectin, a process vital for metastasis to occur.

In vitro invasion, assayed by movement of cells through a layer of ECM, provides a good experimental system to study this component of metastasis. Although the results do not correlate precisely with metastatic potential in vivo, these assays are a good measure of tumour aggressiveness (Thompson et al, 1992; Azzam et al, 1993).

The results reported here show clearly that thrombin potentiates invasion in a breast tumour cell line through an action of its receptor, TR-1. TR-1 is a 7-transmembrane-domain receptor, coupled to $G_i$ and to $G_q$. Stimulation of a cell by thrombin has many effects including: stimulation of phospholipases C, A2 and D; an increase in cytosolic free $Ca^{++}$; activation of protein kinase C, MAP kinases and tyrosine kinases; suppression of cAMP synthesis; stimulation of mitogenesis (Brass, 1995; Brass et al, 1997).

Thrombin is not a chemoattractant in the experiments reported here. Instead, it is a stimulator of invasion because it is added directly to the cells in the invasion chamber. Of the three cell lines, only the MDA-MB231 cells have detectable TR-1 on their surface, and only MDA-MB231 cells respond to thrombin with increased invasion. Thrombin has no effect on chemotaxis with MDA-MB231 cells, so alterations in motility (the capacity of the cells to move) can be ruled out as the mechanism of the thrombin effect. Thrombin does stimulate motility of the MCF-7 cell line, despite the absence of detectable TR-1 on these cells. MCF-7 may have a very low level of TR-1 (undetectable by flow cytometry), or it may respond to thrombin through one of the other protease-activated receptors. For the stimulation of chemotaxis to occur without stimulation of invasion indicates that MCF-7 has little or no ability to digest ECM and permit cell movement.

Expression of TR-1 in these cell lines is independent of ER. Transfection of MDA-MB231 with pHEO (to give MDAL3) did not affect expression of TR-1; the level of TR-1 in MDAL3 cells was not statistically different from that of the parental cells. Expression in MDAL3 cells was not affected by oestradiol in the growth medium. We did find that cell number in the presence of oestradiol was reduced to 70% of the value in the absence of oestradiol. Thus, we confirmed the observation from Rochefort's laboratory (Garcia et al, 1997) that oestrogen slows the growth of the transfected cells.

© Cancer Research Campaign 1999

Whatever thrombin's involvement is in tumour invasion, it occurs through TR-1 because the thrombin effects also occur after TRAP stimulation of the cells. Thus, direct activation by thrombin of matrix metalloproteinases (MMPs) or tissue plasminogen activator is not consistent with our results.

A key question then is whether thrombin and TR-1 can be implicated in invasion through altered expression of MMPs and/or integrins.

MMPs and their role in cancer have been objects of intense research; stromelysin 3 expression (Chenard et al, 1996) and MMP-2 (gelatinase A) activation (Azzam et al, 1993; Bae et al, 1993; Thompson et al, 1994) are associated with increased invasion by breast cancer cell lines. Thrombin effects on MMPs have been studied in vascular cells; thrombin activates pre-existing MMP-2 in vascular endothelial and smooth muscle cells (Zucker et al, 1995; Galis et al, 1997). Evidence for the induction of expression of MMPs by thrombin is not extensive: in vascular endothelial cells, MMP-3 (but not MMP-2) is induced by thrombin (Duhamel-Clerin et al, 1997).

Integrins are cell-surface receptors for ECMs. Alteration in their expression as tumours progress has also been an intensely studied field. The integrin $\alpha 2\beta 1$, which is widely expressed on breast tumour cells, is a receptor for collagen and laminin in less aggressive breast tumour cell lines, but loses its laminin receptor function in more aggressive cell lines such as MDA-MB231 (Maemura et al, 1995). A connection has been made between TR-1 and integrin expression in human colon cancer, rat carcinosarcoma and mouse melanoma (Wojtukiewicz et al, 1995). In these cells, thrombin or the TRAP peptide stimulated cell adhesion to fibronectin. Because integrin $\alpha 3\beta 1$ is a fibronectin receptor (Gui et al, 1997), these results suggest that increased expression of an integrin could result from activation of TR-1.

Thrombin and its receptor, TR-1, are implicated in stimulating invasive activity in MDA-MB231 breast cancer cells. TR-1 expression and thrombin activity appear to be independent of oestrogen receptor expression. TR-1 expression might serve as a prognostic indicator, independent of ER status, of aggressive, invasive breast cancer.

### ACKNOWLEDGEMENTS

This work was aided by the staff and resources of the Wadsworth Center core facilities for cell culture, peptide synthesis, molecular genetics, and molecular immunology. We thank Dr Abigail Snyder-Keller for providing the microscope and NIH Image program for analysis of the data and Dr David Spink for the gift of the MDA-MB436 cell line.

### NOTE ADDED IN PROOF

While this paper was under review, S. Even-Ram et al published a paper with similar conclusions: Even-Ram S, Vziely B, Cohen P, Grisaru-Granovsky S, Maoz M, Ginzburg Y, Reich R, Vlodavsky I and Bar-Shavit R (1998) Thrombin receptor overexpression in malignant and physiological invasion processes. Nature Medicine 4: 909–913.

### REFERENCES

Aragay AM, Collins LR, Post GR, Watson AJ, Feramisco JR, Brown JH and Simon MI (1995) G12 requirement for thrombin-stimulated gene expression and DNA synthesis in 1321N1 astrocytoma cells. *J Biol Chem* **270**: 20073–20077

Arena C, Quirk SM, Zhang YQ and Henrikson KP (1996) Rat uterine stromal cells: thrombin receptor and growth stimulation by thrombin. *Endocrinology* **137**: 3744–3749

Azzam HS, Arand G, Lippman ME and Thompson EW (1993) Association of MMP-2 activation potential with metastatic progression in human breast cancer cell lines independent of MMP-2 production. *J Natl Cancer Inst* **85**: 1758–1764

Bae SN, Arand G, Azzam HS, Pavasant P, Torri J, Frandsen TL and Thompson EW (1993) Molecular and cellular analysis of basement membrane invasion by human breast cancer cells in Matrigel-based in vitro assays. *Breast Cancer Res Treat* **24**: 241–255

Brass LF (1995) Issues in the development of thrombin receptor antagonists. *Thromb Haemost* **74**: 499–505

Brass LF, Manning DR, Cichowski K and Abrams CS (1997) Signaling through G proteins in platelets: to the integrins and beyond. *Thromb Haemost* **78**: 581–589

Chenard MP, O'Siorain L, Shering S, Rouyer N, Lutz Y, Wolf C, Basset P, Bellocq JP and Duffy MJ (1996) High levels of stromelysin-3 correlate with poor prognosis in patients with breast carcinoma. *Int J Cancer* **69**: 448–451

DeMichele MAA and Minnear FL (1992) Modulation of vascular endothelial permeability by thrombin. *Semin Thromb Hemost* **18**: 287–295

Duhamel-Clerin E, Orvain C, Lanza F, Cazenave JP and Klein-Soyer C (1997) Thrombin-receptor mediated increase of two matrix metalloproteinases, MMP-1 and MMP-3, in human endothelial cells. *Arterioscler Thromb Vasc Biol* **17**: 1931–1938

Fidler IJ and Ellis LM (1994) The implications of angiogenesis for the biology and therapy of cancer metastasis. *Cell* **79**: 185–188

Galis ZS, Kranzhofer R, Fenton II JW and Libby P (1997) Thrombin promotes activation of matrix metalloproteinase-2 produced by cultured vascular smooth muscle cells. *Arterioscler Thromb Vasc Biol* **17**: 483–489

Garcia JGN, Pavalko FM and Patterson CE (1995) Vascular endothelial cell activation and permeability responses to thrombin. *Blood Coag Fibrinol* **6**: 609–626

Garcia M, Derocq D, Platet N, Bonnet S, Brouillet JP, Touitou I and Rochefort H (1997) Both estradiol and tamoxifen decrease proliferation and invasiveness of cancer cells transfected with a mutated estrogen receptor. *J Steroid Biochem Mol Biol* **61**: 11–17

Gui GPH, Puddefoot JR, Vinson GP, Wells CA and Carpenter R (1997) Altered cell-matrix contact: a prerequisite for breast cancer metastasis? *Br J Cancer* **75**: 623–633

Kranzhofer R, Clinton SK, Ishii K, Coughlin SR, Fenton II JW and Libby P (1996) Thrombin potently stimulates cytokine production in human vascular smooth muscle cells but not in mononuclear phagocytes. *Circ Res* **79**: 286–294

Maemura M, Akiyama SK, Woods Jr VL and Dickson RB (1995) Expression and ligand binding of a2b1 integrin on breast carcinoma cells. *Clin Exp Metastasis* **13**: 223–235

McNicol A and Gerrard JM (1993) Post-receptor events associated with thrombin-induced platelet activation. *Blood Coag Fibrinol* **4**: 975–991

Nierodzok ML, Bain RM, Liu LX, Shivji M, Takeshita K and Karpatkin S (1996) Presence of the seven transmembrane thrombin receptor on human tumor cells: effect of activation on tumor adhesion to platelets and tumor tyrosine phosphorylation. *Br J Haematol* **92**: 452–457

Pentecost BT (1998) Expression and estrogen regulation of the HEM45 mRNA in human tumor lines and in the rat uterus. *J Steroid Biochem Mol Biol* **63**: 1–9

Price JT, Bonovich MT and Kohn EC (1997) The biochemistry of cancer dissemination. *CRC Crit Rev Biochem Mol Biol* **32**: 175–253

Shapiro SS and McCord S (1978) Prothrombin. In *Progress in Hemostasis and Thrombosis*, vol. 4, p. 177. Grune & Stratton, New York

Thompson EW, Paik S, Brunner N, Sommers CL, Zugmaier G, Clarke R, Shima TB, Torri J, Donahue S, Lippman ME, Martin GR and Dickson RB (1992) Association of increased basement membrane invasiveness with the absence of estrogen receptor and expression of vimentin in human breast cancer cell lines. *J Cell Physiol* **150**: 534–544

Thompson EW, Yu M, Bueno J, Jin L, Maiti SN, Palao-Marco FL, Pulyaeva H, Tamborlane JW, Tirgari R and Wapnir I (1994) Collagen-induced MMP-2 activation in human breast cancer. *Breast Cancer Res Treat* **31**: 357–370

Tora L, Mullick A, Metzger D, Ponglikitmongkol M, Park I and Chambon P (1989) The cloned human estrogen receptor contains a mutation which alters its hormone binding properties. *EMBO J* **8**: 1981–1986

Vittet D and Chevillard C (1993) Thrombin interactions with cells of the megakaryocytic lineage. *Blood Coag Fibrinol* **4**: 759–768

Wojtukiewicz MZ, Tang DG, Ciarelli JJ, Nelson KK, Walz DA, Diglio CA, Mammen EF and Honn KV (1993) Thrombin increases the metastatic potential of tumor cells. *Int J Cancer* **54**: 793–806

© Cancer Research Campaign 1999 

Wojtukiewicz MZ, Tang DG, Ben-Josef E, Renaud C, Walz DA and Honn KV (1995) Solid tumor cells express functional 'tethered ligand' thrombin receptor. *Cancer Res* **55**: 698–704

Zacharski LR (1996) Blood coagulation factors in tumor tissue: implications for cancer therapy. In *Malignome und Haemostase*. Spanuth E (ed.), p. 134. Springer Verlag: Berlin

Zacharski LR and Costantini V (1995) Blood coagulation activation in cancer: challenges for cancer treatment. *Hamostaseologie* **15**: 14–20

Zacharski LR, Wojtukiewicz ME, Costantini V, Ornstein DL and Memoli VA (1992) Pathways of coagulation/fibrinolysis activation in malignancy. *Semin Thromb Hemost* **18**: 104–116

Zacharski LR, Memoli VA, Morain WD, Schaeppl JM and Rousseau SM (1995) Cellular localization of enzymatically active thrombin in intact human tissues by hirudin binding. *Thromb Haemost* **73**: 793–797

Zucker S, Conner C, DiMassmo BI, Ende H, Drews M, Seiki M and Bahou WF (1995) Thrombin induces the activation of progelatinase A in vascular endothelial cells. *J Biol Chem* **270**: 23730–23738

 *© Cancer Research Campaign 1999*
